# Supplementary material for: Image Filtering to Improve Maize Tassel Detection Accuracy Using Machine Learning Algorithms
Source: Sensors (Basel). 2024 Mar 28;24(7):2172. doi: 10.3390/s24072172 (PMC11013961; doi:10.3390/s24072172)
Supplement: Supplementary file 1 [file sensors-24-02172-s001.zip › sensors-2898337-supplementary.pdf]

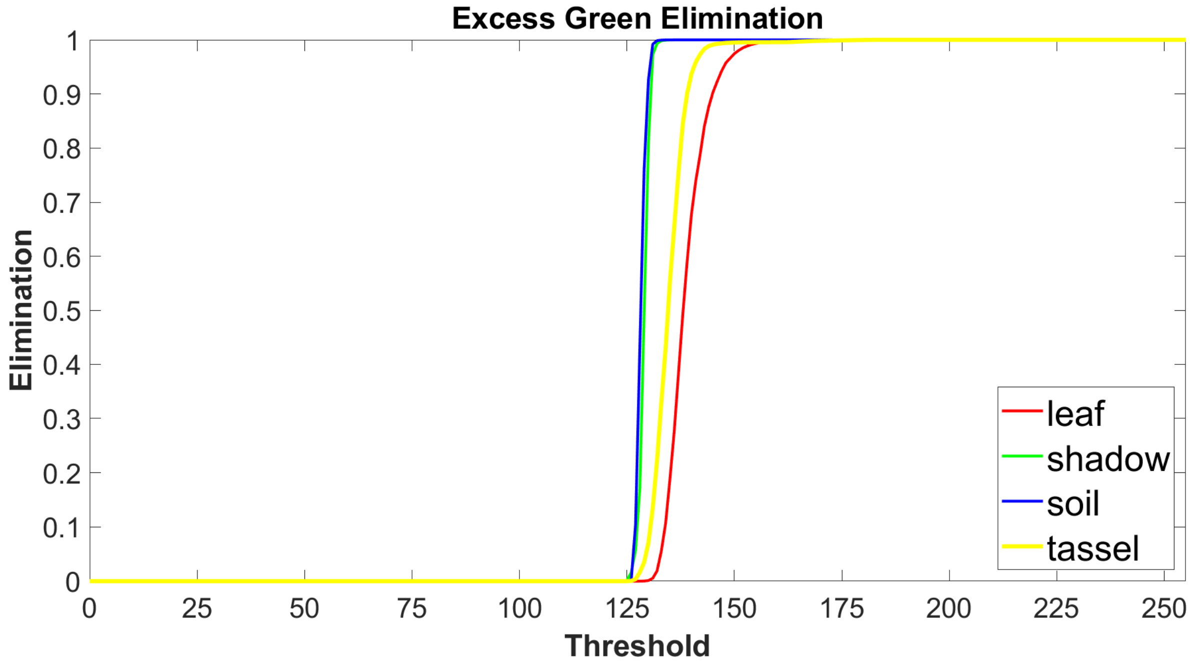

Figure S1: The elimination chart of the Excess Green Index. This index eliminates bare soil and shadows on the ground at almost identical thresholds, while pixels representing tassels and foliage tend to be eliminated at higher thresholds. There is a noticeable gap between the thresholds at which soil and shadows are eliminated and the thresholds at which tassels are eliminated, meaning the image dataset generally can be filtered using this approach without significantly removing the tassels.

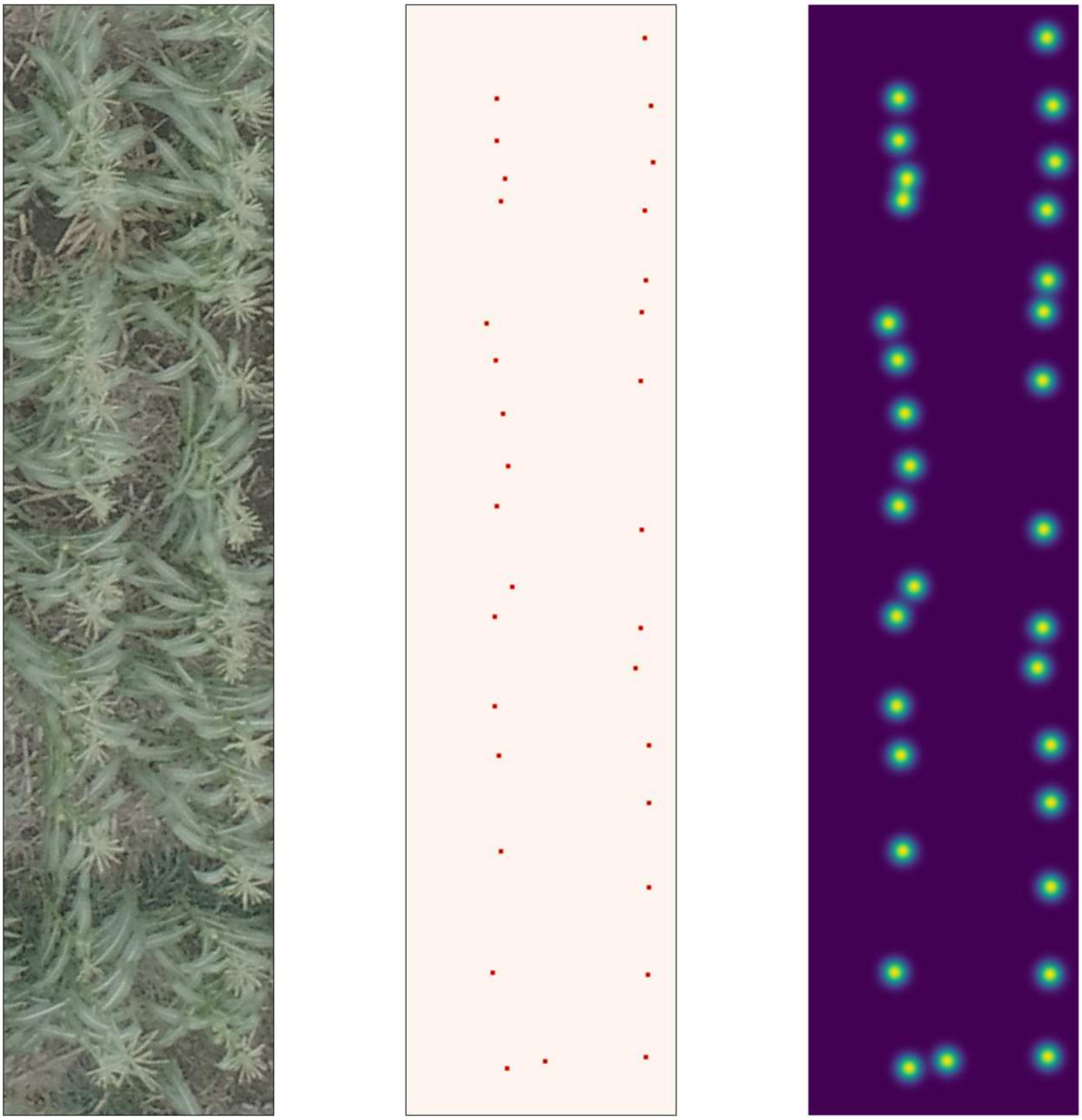

Figure S2: Density map obtained from TasselNet using a test image. The left panel shows the original image captured from the UAV device. The middle panel is the point-referenced map identifying the location of tassels in the original image. The right panel shows the density map obtained by smoothing the point-referenced map using a bivariate Gaussian smoothing kernel.

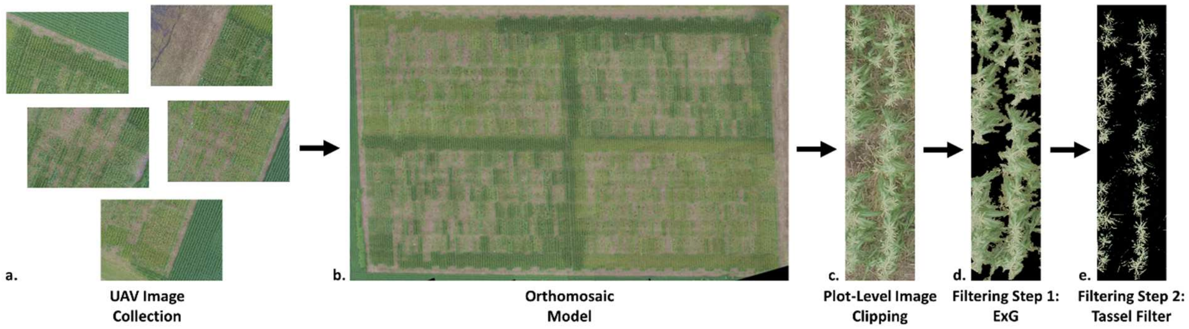

Figure S3: Image extraction and filtering pipeline. The raw UAV images (**a**) are used to generate an orthomosaic model (**b**) using the software Phenix. This model then allows the plot-level images to be clipped from the original UAV images (**c**). Once extracted, the plot-level images can then be used to train the detectors as they are, without filtering, or they may be filtered first. Filtering is a two-step process. First, the clipped images are filtered using the Excess Green Index to remove non-foliage pixels in the images (**d**). These images are then further filtered using the formula we have described to also remove the foliage and only leave behind pixels associated with tassels (**e**).

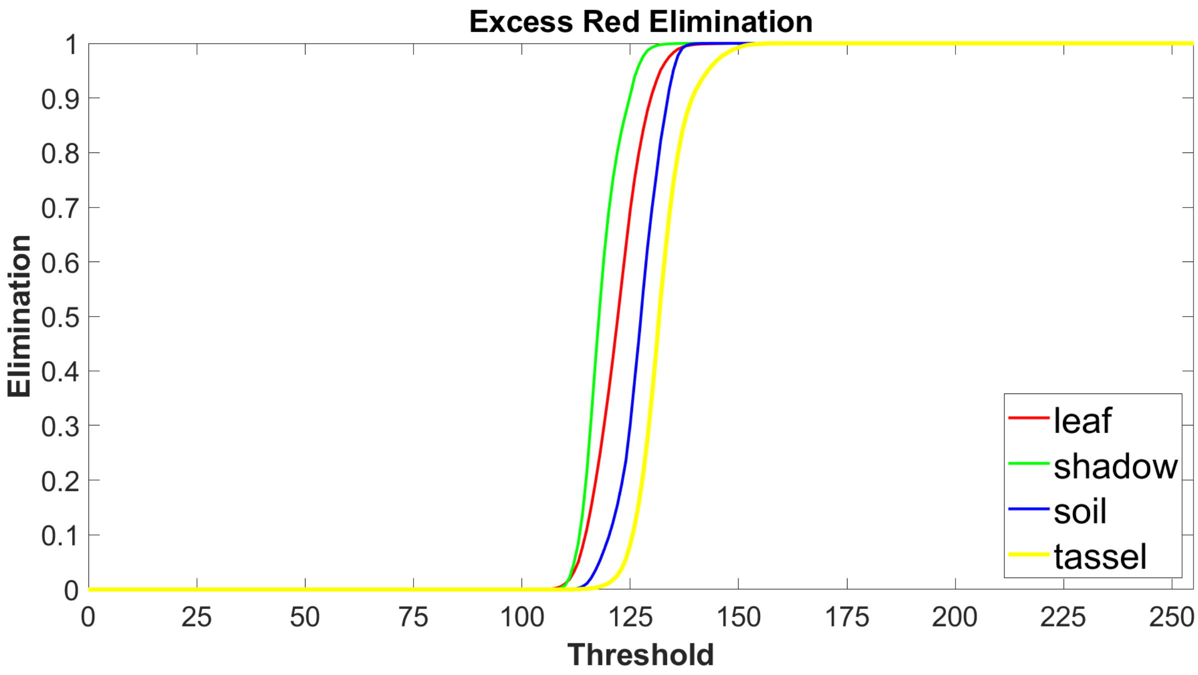

Figure S4: The elimination chart of the Excess Red Index.

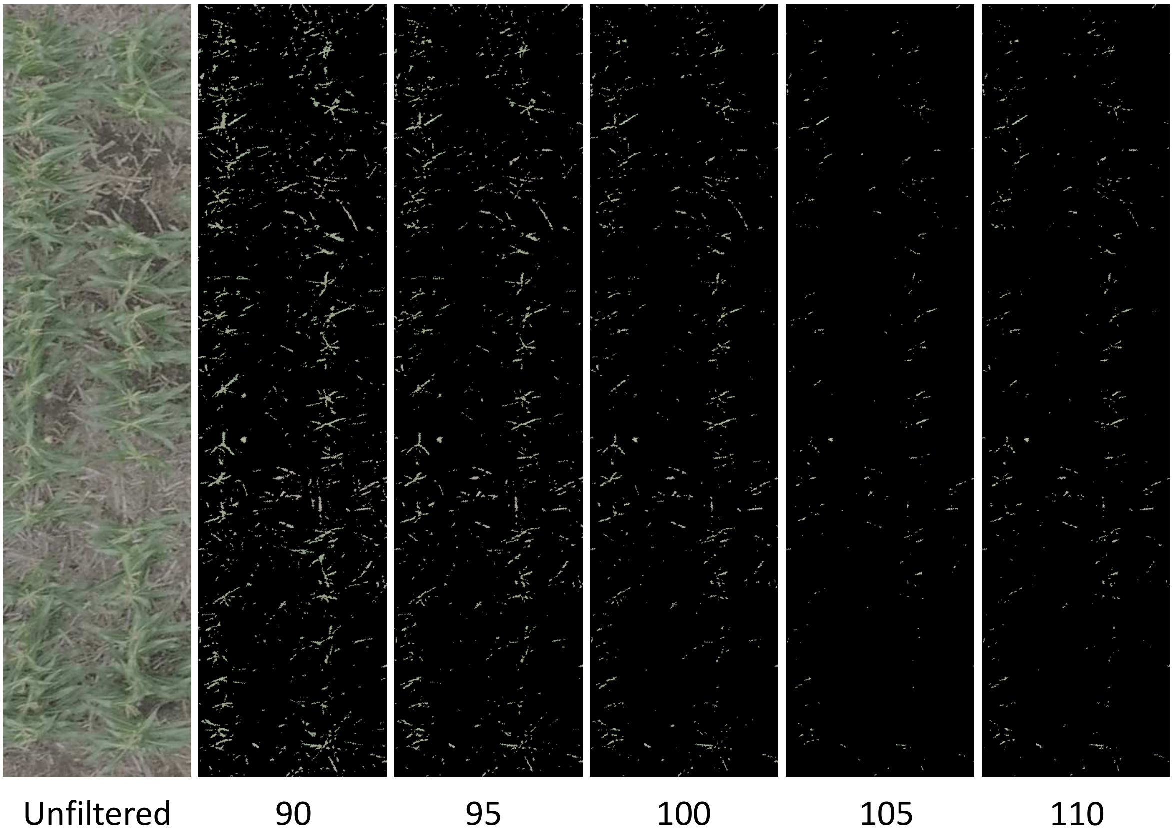

Figure S5: An example showing the tassel filter at thresholds of 90 to 110, starting with an unfiltered image (far left).

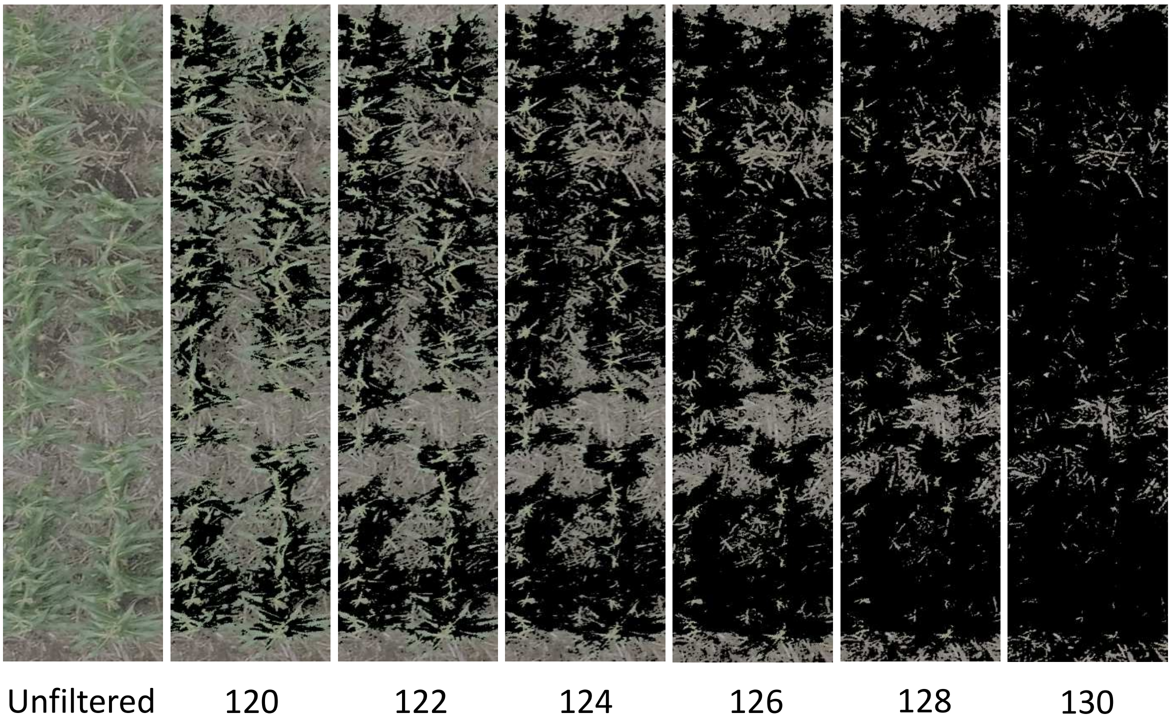

Figure S6: An example showing the difficulty the Excess Red Index has in distinguishing between soil and tassels in unfiltered images. The filtered images range from thresholds of 120 to 130.

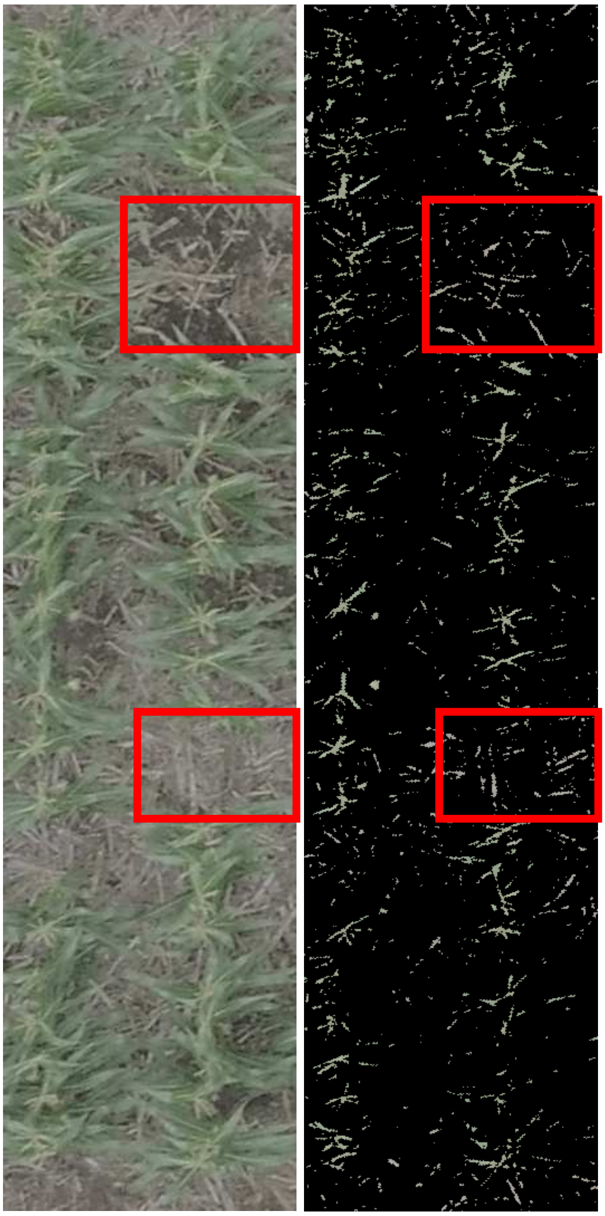

Figure S7: An example showing how the tassel filter has some difficulty distinguishing between tassels and debris in unfiltered images. The right image was filtered at a threshold of 90.
